# Supplementary material for: Analysis of football research trends using text network analysis
Source: PLoS One. 2024 Apr 18;19(4):e0299782. doi: 10.1371/journal.pone.0299782 (PMC11025783; doi:10.1371/journal.pone.0299782)
Supplement: Attachment — Submitted filename: Response to reviewers (Rebuttal Letter).docx [file pone.0299782.s002.docx]

**'Response to Reviewer 1'**

\#### Overview
The manuscript provides a comprehensive analysis of football research trends over the last thirty years using a text network analysis. The approach is innovative and the topic is relevant to the field.

\#### Major Comments for Revision

1. **Methodology Clarity**: The methodology section, while thorough, could benefit from clearer explanations of the text network analysis process. A step-by-step breakdown would enhance the reader's understanding.

- The procedure of study is newly explained in method section, and the order of the text network analysis process explained a step-by-step.

2. **Data Representation**: The manuscript presents a vast amount of data. However, the current visual representations (graphs, tables) could be improved for better clarity and impact.

- A new figure (figure 1) is presented in method section to clearly explain the process data collected and help for reader to understand.

3. **Comparative Analysis**: The study primarily focuses on keyword trends. Including a comparative analysis with other sports or academic disciplines could provide a richer context.

- The comparative analysis is explained in discussion section as a limitation and future suggestions.

4. **Discussion of Limitations**: While some limitations are mentioned, a more detailed discussion regarding the constraints of the methodology and potential biases in keyword selection is needed.

- The more detailed limitations are explained in discussion section.

5. **Implications and Future Research**: Expand on the practical implications of the findings. Also, suggest specific areas for future research based on the trends observed.

- The suggestions are explained for future research in discussion section.

6. **References and Citations**: Ensure all references are up-to-date and relevant. Some citations seem dated considering the fast-paced changes in the field.

- References are revised e.g. removed irrelevant articles and added up-to-date and relevant articles in reference section.

**'Response to Reviewer 2'**

Reviewer #2: Dear Dr Jongwon Kim,

Thank you for the opportunity to review this paper. I have read the paper and made some comments based on my observations which are highlighted in this document.

It is clear you have conducted a large amount of research using some advanced techniques to identify the appropriate papers to be used within this study and the commitment to do so should be congratulated. However, from a scientific perspective, the methodology and statistical analysis techniques have not been explained thoroughly enough and with sufficient detail within the manuscript thus, the experimentation would be difficult to replicate.

For example, while formulas are provided for degree centrality and Eigenvector centrality, the components of these formulas are not fully explained. Each formula uses the ‘total number of nodes’ as a measure but an explanation of what the nodes are specifically is not included within the text which is confusing for the reader as it is the first time that nodes are mentioned in the manuscript. Furthermore, within the clustering analysis section, the terms ‘modularity coefficient’ and the ‘greedy heuristics algorithm of the hard clustering method’ are both mentioned without explanation or reference. In addition, explanations are not provided for other terms such as the Gini Coefficient, Ochiai coefficient and Salton coefficient either.

While the concept of using different analysis methods (network analysis) compared to more traditional review methods is an interesting one, I am unsure on the value the information from this manuscript provides to the scientific community. It is interesting to see how research trends have changed throughout the last 30 years, but this manuscript does not provide comprehensive insights into the reasoning behind these trends and how these may shape further research. The discussion needs further development to highlight the benefits of this study and to provide more in-depth insights on its value for the reader. The discussion needs to draw more on previous research to build these points.

I believe this manuscript has not been written with a proficient standard of the English language to be considered for publication in Plos One. There are frequent grammatical, punctual and tense based errors throughout the manuscript that make it difficult to read in places and in some instances, it does not fully make sense. These issues require attention before this manuscript can be considered ready for publication.

I hope the reasons highlighted in this document have provided some clarity as to how a decision was made regarding this manuscript. Please do not be disheartened by these comments as you have clearly developed a high-level skillset to conduct this research, but the manuscript needs further development before it is ready to be submitted for publication.

- 1. The explanation of the nodes included within the text is added in building network section.
- 2. The definition of terms ‘modularity coefficient’ and the ‘greedy heuristics algorithm of the hard clustering method’ are explained with references.
- 3. The definitions of terms ‘Gini Coefficient’, ‘Ochiai coefficient’ and ‘Salton coefficient’ are explained with references.
- 4. A more discussion with comprehensive insights with these findings and benefits of this study is explained in discussion section.
- 5. This manuscript is re-written from Native English Language Speaker to revise grammar mistake and make it easy for reader to understand with fully make sense.

**'Response to Reviewer 3'**

Reviewer #3: Author should add the Temporal Analysis:
Analyzing the temporal evolution of football research trends allows us to trace the emergence and decline of specific topics over time. This can provide insights into the dynamic nature of football-related scholarship.

- The temporal analysis including the change of amount and subject is added in result section.

Thank you for the valuable feedback from the reviewers. I have incorporated the suggestions from the reviewers, and the paper has been improved accordingly. Please review the revised sections. Thank you very much.
