# Supplementary material for: Analysis of football research trends using text network analysis
Source: PLoS One. 2024 Apr 18;19(4):e0299782. doi: 10.1371/journal.pone.0299782 (PMC11025783; doi:10.1371/journal.pone.0299782)
Supplement: Attachment — Submitted filename: Response to reviewers (Rebuttal Letter).docx [file pone.0299782.s003.docx]

**'Response to Reviewer 1'**

Reviewer #1: I have reviewed the revised version of "Analysis of football research trends using text network analysis" for Plos One. The authors have made commendable improvements based on the initial feedback. However, I suggest an essential enhancement: the manuscript should draw connections with key scientometric studies that have utilized Social Network Analysis (SNA).

To strengthen the manuscript's relevance and theoretical grounding, I recommend referencing the following studies:

1. <https://doi.org/10.1016/j.eswa.2022.117853>
2. <https://doi.org/10.1111/sms.14019>
3. <https://doi.org/10.1016/j.joi.2011.05.007>

These references are pivotal in aligning the study with foundational work in the field and clarifying its contribution to scientometric research.

Thank you for the opportunity to review this manuscript. I believe these additions will significantly enhance its impact.

Sincerely,

- 1. I have added the pivotal references in this field including papers reviewer recommended.
- 2. I have added additional explanation about Social Network Analysis (SNA) to help readers to understand the conception.
- 3. I have added related previous research paper with my topic to compare the methodology and results.
- 4. I have added comparison and discussion about previous literature.

**'Response to Reviewer 3'**

Reviewer #3: proposed changes are well incorporated, So, it is recommended for acceptance and for further publishing process.

- Thank you for the valuable feedback to enhance this paper.

Thank you for all the valuable feedback from all reviewers. I have incorporated the suggestions from the reviewers, and the paper has been improved accordingly. Please review the revised paper. Thank you very much.
